# Profiles of current COVID-19 vaccines

**DOI:** 10.1007/s00508-021-01835-w

**Published:** 2021-03-16

**Authors:** Franz X. Heinz, Karin Stiasny

**Affiliations:** Center for Virology, Medical University of Vienna, Kinderspitalgasse 15, 1090 Vienna, Austria

## Preface

We currently experience a milestone phase in the history of vaccination, with first effective coronavirus disease 2019 (COVID-19) vaccines authorized for human use within only approximately 1 year after the discovery and global expansion of a new human virus, severe acute respiratory syndrome coronavirus 2 (SARS-CoV-2). Some of these vaccines were produced by established conventional approaches, whereas others reflect breakthroughs of novel technologies that are based on at least three decades of scientific development and have now reached the market of mass vaccination for the first time. In this perspective article, we discuss the specific features of the viral antigen used as an immunogen and present the basic concepts behind current mRNA, adenovector, inactivated whole-virus and subunit vaccines, describing similarities and differences. We also address the structural basis of antigenic variation exhibited by newly emerging viral variants, which may pose new challenges to the immunoprophylaxis of COVID-19 by vaccination.

## The viral spike protein

**Fig. 1:**
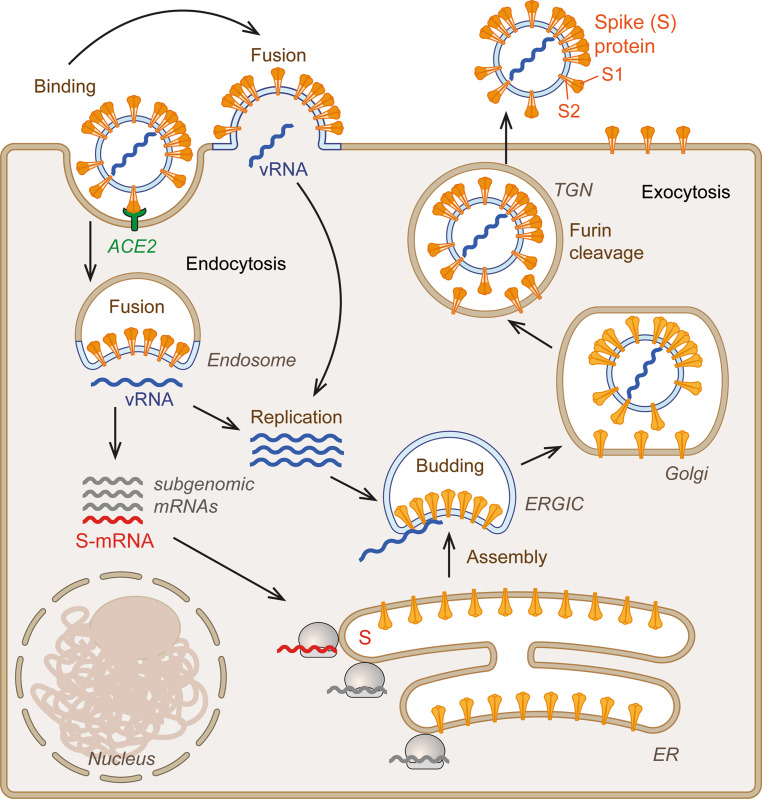
Simplified life cycle of SARS-CoV‑2. The S protein of SARS-CoV‑2 binds to the cellular receptor angiotensin-converting enzyme 2 (ACE2). Cell entry occurs either through fusion at the plasma membrane or with an endosomal membrane after uptake by receptor-mediated endocytosis, resulting in release of the viral RNA (vRNA) into the cytoplasm. Genome replication and expression lead to the formation of genomic RNA as well as a set of subgenomic mRNAs, one of which encodes the S protein (*highlighted in red*). This mRNA is translated at ribosomes associated with the endoplasmic reticulum (ER), and newly synthesized S is transported into the lumen of this compartment. Formation of new virus particles occurs by budding into the lumen of the endoplasmic reticulum (ER)-golgi intermediate compartment (ERGIC). Virions are released from the infected cell through exocytosis. During transport, the spike protein is cleaved into S1 and S2 in the trans-Golgi network (TGN) by the cellular protease furin

**Fig. 2:**
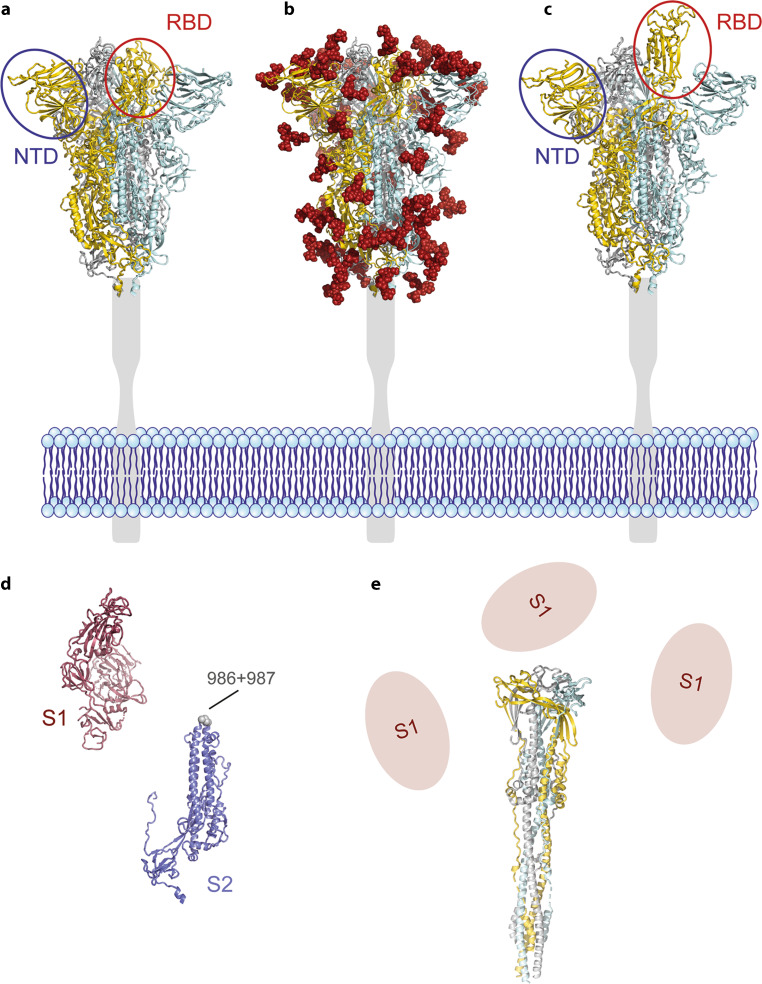
Structural organization of the spike protein of SARS-CoV‑2. **a**, **b** Cartoon representations (*side view*) of the trimeric spike (composed of three identical S proteins) in its prefusion conformation with the receptor-binding domains (RBD) down (closed conformation). In panels (**a**) and (**c**) one N-terminal domain N‑terminal domain (NTD) and one RBD are encircled in *blue* and *red*, respectively. In panel (**b**) the glycans present at the surface of the spike are shown as *red spheres* (modeled with GlyProt [10]). **c** Cartoon representation (*side view*) of the trimeric spike in its prefusion conformation with one receptor-binding domains (RBD) up (open conformation). The RBD is encircled in red, the N‑terminal domain (NTD) in *blue*. Color code of panels (**a**–**c**): The three protomers in S are colored in *gold*, *gray* and *light blue*. The stalk and transmembrane domain of S are depicted as *light gray bars*, the viral membrane in *blue*. **d** Cartoon representation of the two subunits of S (S1—*red* and S2—*blue*) in their prefusion conformation, separated for clearness. The positions of the two stabilizing mutations in S2 (amino acid positions 986, 987) are shown as *grey spheres*. **e** Cartoon representation (*side view*) of the S2 trimer in its postfusion conformation. The three protomers are colored in *gold*, *gray* and *light blue*. Dissociated S1 subunits are shown in *light red*. The following structures from the protein data bank (PDB) were used for the graphics: PDB ID 6ZGI, prefusion conformation with the RBDs down [11]; PDB ID 6ZGG, prefusion coformation with one RBD up [11]; PDB ID 6XRA, postfusion conformation [12].

All COVID-19 vaccines that have completed phase 3 clinical trials and are currently applied in different countries of the world use the entire viral spike protein (S) as an immunogen (Figs. 1 and 2). The mode of delivery to the vaccinee and its presentation to the immune system, however, are fundamentally different in these vaccines, as is explained in more detail in the following vaccine-specific sections. The focus on S is based on the fact that antibodies directed to its native conformation are potent inhibitors of virus entry and therefore can neutralize viral infectivity (Fig. 1; [1–3]). Importantly, the S protein of coronaviruses is prone to undergo dramatic structural changes that occur at the membrane fusion stage of the viral life cycle and are essential for the infection process (Figs. 1 and 2) (reviewed in [4]). The inherent structural instability of this key antigen has been an important aspect in the design of vaccines, because loss of its native conformation may lead to the induction of antibodies with lower neutralizing potency.

The viral spike is a complex of three identical, membrane-anchored S proteins, which are highly glycosylated and comprise 1273 amino acids each (Fig. 2; [5–7]). Recent cryo-electron microscopical analyses of viral particles provide evidence that a relatively low number of spikes (on average only 24) are irregularly distributed in the viral membrane, corresponding to a spike density that is about tenfold lower than that of influenza A virus [8]. Molecular hinges in the membrane-proximal part of S allow tilting in all directions and provide considerable flexibility to the molecule [8, 9].

In the course of natural cellular infection (Fig. 1) (reviewed in more detail in [13]), S is synthesized from one of the viral mRNAs into the lumen of the endoplasmic reticulum (ER) as a membrane-associated protein and transported to the endoplasmic reticulum-Golgi intermediate compartment (ERGIC). The S protein is retained due to an ER retrieval signal and interactions with the viral membrane protein M [14], but some molecules of S are also transported to the plasma membrane [15]. Virus assembly occurs in the ERGIC, and newly formed viral particles are released by exocytosis (reviewed in [15]). In the course of secretion, the S protein is cleaved by the cellular protease furin into the membrane-associated S2 part and the distal S1 part, which however remain associated through non-covalent interactions (Figs. 1 and 2). Both parts provide essential functions during viral entry. S1 mediates binding to ACE2 on target cells through its receptor-binding domain (RBD), which oscillates between an “up” and “down” conformation in the trimeric spike ([5, 6, 16], Fig. 2a,c). Receptor interaction is possible only in the “up” conformation. S2 mediates fusion of the viral membrane with either the plasma membrane or endosomal membranes (Fig. 1), resulting in the release of the RNA genome into the cytoplasm (reviewed in [4]). During this process, S1 falls off and S2 adopts a radically different conformation, the postfusion structure, which is also a trimer (Fig. 2d,e; [12]). Understanding the specific structural properties of the spike protein, especially its built-in instability, is important for the use of S as an immunogen in vaccines.

Untoward effects because of conformational instability of the vaccine antigen have been experienced with a formalin-inactivated vaccine against respiratory syncytial virus (RSV) developed in the 1960s [17]. The vaccine was shown to be responsible for enhanced disease upon natural infection in the following season. This failure was directly related to the fact that the procedure for preparing the vaccine had converted the envelope fusion proteins (responsible for inducing protective neutralizing antibodies) into their postfusion conformation [18]. Therefore, the antibody response was only poorly neutralizing and consisted primarily of antibodies binding to the conformationally flipped protein [19]. This negative experience resulted in elegant, structure-based work that finally led to the stabilization of the protein in its prefusion conformation by the engineering of specific mutations that prevent its undesirable conversion into the postfusion structure [20]. Because of the structural similarities of RSV and coronavirus envelope proteins (both class I fusion proteins, [21]), the same principle of mutational protein stabilization could also be applied to coronavirus S proteins, first to those of SARS‑1 and Middle East respiratory syndrome (MERS) viruses [22, 23] and more recently also to the S protein of SARS-CoV‑2 [5, 6]. Specifically, two consecutive prolines are engineered into a specific site of the S2 moiety of S (Fig. 2d) that prevent the undesirable refolding of S2. Several of the current COVID-19 vaccines make use of this technology for antigen stabilization, as indicated in the specific sections.

Recent studies demonstrated that most neutralizing antibodies (ca. 90%) in human postinfection sera are specific for the RBD [1] and a number of potently neutralizing RBD-specific human monoclonal antibodies have been described in the literature (reviewed in [24]). Despite the apparent immunodominance of the RBD [1], the N‑terminal domain (NTD), which, like the RBD is exposed in the prefusion conformation of the spike (Fig. 2a,c), can also induce extremely potent neutralizing antibodies [25, 26]. Currently, there is no generally accepted in vitro correlate of protection, and the situation is further complicated, because such correlates may differ between different categories of vaccines. The induction of S‑specific virus neutralizing antibodies, however, is considered to be of key importance, although other immune functions (especially those involving specific CD4 and CD8 cells) may contribute to protection [27].

## mRNA vaccines

**Fig. 3:**
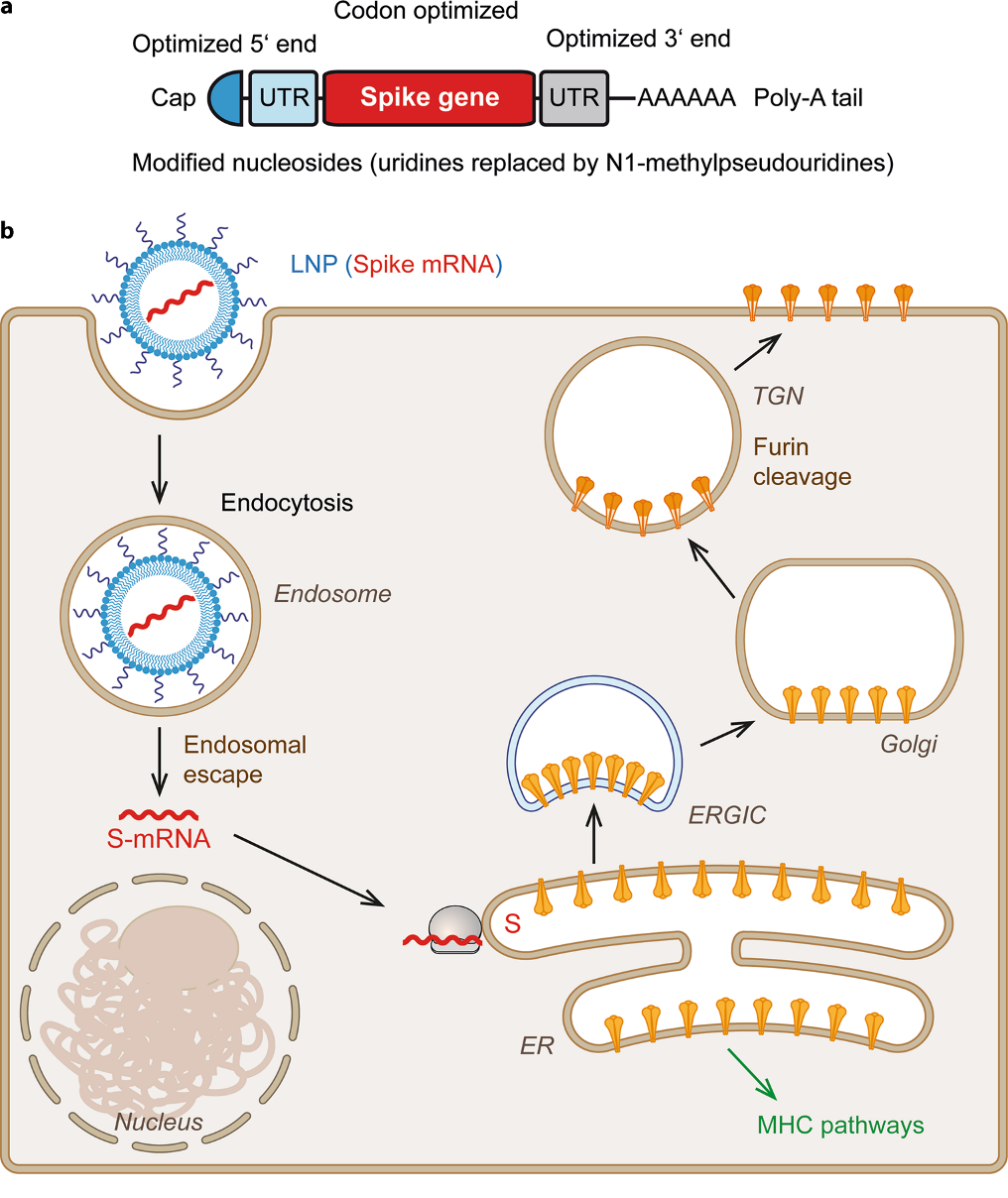
mRNA vaccines. **a** Schematic representation of the vaccine mRNA (*UTR* untranslated region). **b** Schematic diagram of mRNA vaccine-mediated expression of the S protein in transduced cells. mRNA coding for full-length S, encapsulated into a lipid nanoparticle (LNP), enters the cell by endocytosis (or direct fusion with the plasma membrane, not shown). After endosomal escape into the cytoplasm, the S‑mRNA is translated at ribosomes associated with the endoplasmic reticulum (ER) and newly synthesized S is transported into the lumen of this compartment, similar to the process occurring in natural infection (Fig. 1). Further transport occurs via the exocytic pathway, leading to expression of S at the plasma membrane. The intracellularly synthesized protein is also degraded and enters the MHC (major histocompatibility complex) I and II pathways (not shown)

Recently, two COVID-19 mRNA vaccines (from Biontech/Pfizer and Moderna) have been authorized in different countries (including the USA and the EU) after successful clinical trials and are already in widespread use [28, 29]. The two products contain a nucleoside-modified mRNA, encoding the sequence of the full-length S protein with two stabilizing proline mutations in S2 (Figs. 2 and 3a) to preserve the native prefusion conformation. Both vaccines use lipid nanoparticles for delivery. Because of their fragility, these vaccines require storage at −70 °C (Biontech [Biontech, Mainz, Germany]/Pfizer [Pfizer, New York, NY, USA]) or −20 °C (Moderna [Moderna, Cambridge, MA, USA]) and contain 30 µg and 100 µg RNA, respectively. From the data of phase 3 clinical trials, protection rates against disease were as high as 95% and 94.1%, respectively, after 2 vaccinations, with tolerable side reactions [30, 31]. This success represents an enormous leap forward in the establishment of a novel technology at the leading edge of the COVID-19-vaccine landscape.

The concept of using RNA as a form of genetic vaccine is not new and was already developed in the early 1990s, based on the demonstration that in vitro transcribed mRNA is expressed in vivo after direct injection into mouse muscle [32]. The principle appears as elegant as straightforward. An mRNA encoding the vaccine antigen is injected, enters cells and the cellular protein translation machinery produces the appropriate antigen, followed by an effective immune response (Fig. 3). Although the idea caused considerable excitement early on, practical implementation was hampered by a number of technical obstacles, including RNA degradation, inefficient entry into cells and suboptimal translation for producing the antigen in the vaccinee, as well as excessive inflammatory responses caused by the recognition of foreign RNA through receptors of the innate immune system [33]. The delivery and stability problem was solved by the development of lipid-based carriers [34], such as the lipid nanoparticles (LNPs) used in the current RNA vaccines (Fig. 3). The chemical composition of these carriers is documented [28, 29], although the formulation process during production may rely on a number of details representing know-how of the producing companies. At least as important as the application of LNPs for delivery was the modification of the RNA in such a way that innate responses are dampened (to avoid intolerable side reactions and the associated restriction of protein translation), without losing the intrinsic adjuvant activity of RNA and efficiency of antigen synthesis [35–37]. These changes are a scientific topic on their own and include the codon optimization of the coding sequence, modifications of untranslated regions both at the 5’ and 3’ ends of the RNA to increase stability and translation efficiency as well as the discovery that introduction of modified nucleotides can significantly contribute to the right balance required for an efficacious and well-tolerated RNA vaccine ([36–38]; Fig. 3a). As a consequence of this research, uridines are completely replaced by N1‑methylpseudouridines in both the Biontech/Pfizer and the Moderna vaccines [39, 40]. An mRNA vaccine without uridine replacement and prolonged stability at 4 °C is manufactured by the German company Curevac (Curevac, Tübingen, Germany) [41] and currently undergoes testing in a phase 2b/3 clinical trial [42]. Even without going into the details of the RNA modifications indicated in Fig. 3a, it is apparent that several scientific breakthroughs were required to overcome all of the hurdles associated with RNA delivery and to bring RNA vaccines to the current stage of practical application.

## Adenovector vaccines

Like with RNA vaccines, the sudden global need for prophylactic mass immunization against COVID-19 was an enormous driving force for pushing forward previously established adenovector vaccine platforms [43]. Meanwhile, several vaccines based on different non-replicating adenovirus vectors and the full-length S protein have completed phase 3 clinical trials and are used for vaccination campaigns in many countries after approval by national and international authorities. These vaccines include products of the Gamaleya Institute in Moscow (Sputnik V) [44, 45], University of Oxford/AstraZeneca (AstraZeneca, Cambridge, UK) (ChAdOx1-S/AZD1222) [46], the Beijing Institute of Biotechnology/CanSino (CanSino Biologics, Tianjin, China) [47] and Janssen Pharmaceutica (Janssen Pharmaceutica [pharmaceutical company of Johnson & Johnson], Beerse, Belgium) [48].

**Fig. 4:**
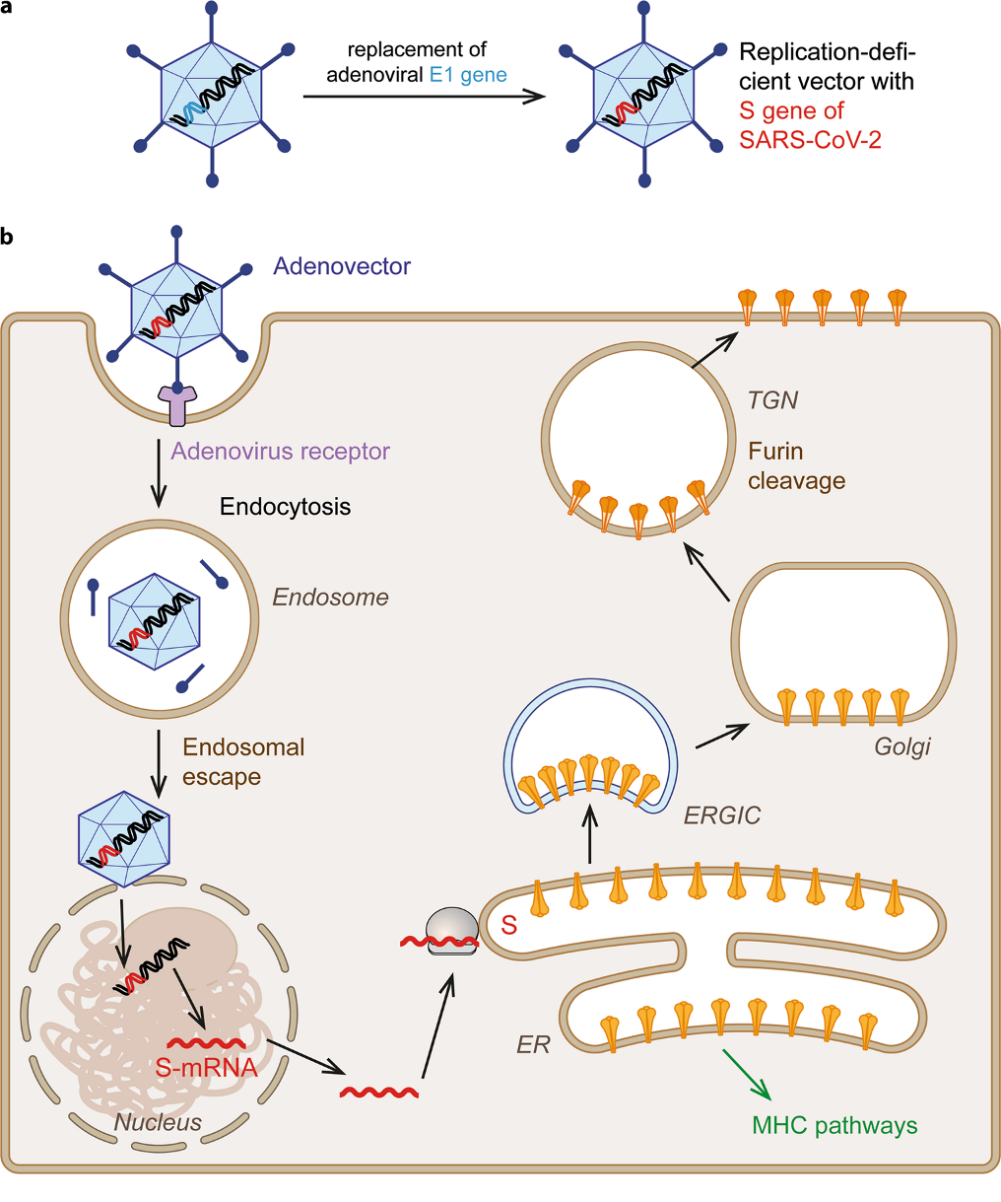
Adenovector vaccines. **a** Principle of non-replicating adenovector vaccine. **b** Schematic diagram of adenovector vaccine-mediated expression of the S protein in transduced cells. An adenovector containing the full-length S gene as part of the viral DNA enters the cell by receptor-mediated endocytosis. After endosomal escape into the cytoplasm, the capsid traffics to the nucleus. The adenoviral DNA is transferred into the nucleus, where it remains extrachromosomal and gives rise to the transcription of an S‑specific mRNA. Similar to the processes after natural infection (Fig. 1) and mRNA vaccination (Fig. 3) the S mRNA is translated at ribosomes associated with the endoplasmic reticulum (ER) and newly synthesized S is transported into the lumen of this compartment. Further transport occurs via the exocytic pathway, leading to expression of S at the plasma membrane. The intracellularly synthesized protein is also degraded and enters the major histocompatibility complex (MHC) I and II pathways (not shown)

The idea of using adenoviruses as vectors was originally developed for gene therapy [49] and only later applied to vaccines, building on their capacity to induce potent innate and adaptive immune responses [43, 50]. In the current COVID-19 vaccines and for reasons outlined, different adenoviruses are used as vectors but the basic principle of production platforms and mechanism of action is the same (Fig. 4). The gene for the SARS-CoV‑2 S protein is synthesized as a DNA and engineered into the DNA genome of adenoviruses, replacing an adenovirus gene (E1) that is essential for virus replication (Fig. 4a). Through this manipulation, the adenovirus can no longer replicate and cannot give rise to a full infectious cycle (it is therefore referred to as non-replicating viral vector), but it can still enter cells and express the inserted foreign gene to produce the coronavirus S protein (Fig. 4b). In the course of the single round of infection after vaccination, adenovirus vector particles recognize specific cellular receptors, are internalized by receptor-mediated endocytosis and transported to the nuclear membrane where the viral DNA is transferred into the nucleus. The DNA remains extrachromosomal (like in natural adenovirus infections), but the inserted gene for the S protein is transcribed into a corresponding mRNA that is transported to the cytoplasm, followed by the same sequential steps of S protein production and intracellular transport as in the case of natural infection and mRNA vaccination (compare Figs. 1, 3b and 4b).

For adenovirus-vector vaccines to be effective, high doses of vector particles have to be applied. Upon entry into cells, these particles are recognized by sensors of innate immunity [43, 50] and give rise to the release of cytokines and chemokines that are responsible for side reactions associated with vaccination. This intrinsic reactogenicity has the advantage of a built-in adjuvant effect (similar to that of mRNA vaccines), but at the same time limits the dose that can be used for vaccination. Dose finding was therefore an important aspect of human immunization studies to find the right balance between immunogenicity and reactogenicity. As indicated in the reports of phase 1, 2, and 3 clinical trials, the number of viral particles in the current adenovector vaccines is 5 × 10^10^ (AstraZeneca [46], Janssen [48]) or 1 × 10^11^ (Sputnik V [44]). This is a substantial mass of adenovirus antigen contained in the vaccine (representing approximately 9 µg or 18 µg of adenovirus protein; calculation based on approximately 100 million amino acids composing the adenovirus capsid [51]). The amount of adenoviral antigen is thus in the range of the antigen content in conventional inactivated or subunit viral vaccines, resulting in an adenovirus-specific immune response in parallel to that against the S protein in the course of COVID-19 adenovector immunization.

Historically, human adenovirus 5 (hAd5) has pioneered the development of the adenovector platform [43, 50]. This vector is used in the Chinese CanSino vaccine and in one of the two components of Sputnik V [44, 47]. Despite its robust immunogenicity, human populations have substantial seropositivity against adenovirus 5 (e.g. 61% in Europe, 65–100% in Africa; [52]), which can dampen the response to the vaccine antigen as compared to individuals without pre-existing immunity [50, 53]. To avoid this potential disadvantage, other serotypes of adenoviruses have been established as vector platforms [54, 55]. The vaccine manufactured by Janssen (Johnson&Johnson) makes use of human adenovirus 26 (hAd26, global seroprevalence only 5.4–17.8% [52]) and recent results from phase 3 clinical trials indicate an efficacy in preventing moderate and severe COVID-19 at 28 days postvaccination of 72% in the USA, 66% in Latin America, and 57% in South Africa [56]. Based on these data the company has applied for an emergency use authorization in the USA [28]. The same hAd26 vector is also applied as a first dose in the two-component Sputnik V vaccine in combination with adenovirus 5, which is in use in Russia and other countries [57] and has an efficacy of 91.6% in a recently published phase 3 clinical trial [45].

The problem of pre-existing immunity is lowest with the Oxford/AstraZeneca vaccine, because it is based on a chimpanzee adenovirus that has very low seropositivity except for certain parts of Africa [52, 58]. Phase 3 clinical trials have indicated an overall efficacy of 70.4% in preventing COVID-19, and substantial efficacy was already achieved after the first dose [46]. The vaccine has received an emergency use authorization in the UK and was recently licensed in the European Union [29]. Irrespective of pre-existing immunity, however, all adenovector vaccines have to deal with the issue of vaccination-induced vector immunity, which may become a problem for booster vaccinations after several doses [59]. Therefore, the exploitation of prime-boost strategies with different vectors are being investigated, similar to the strategy used in the Sputnik V vaccine (i.e. hAd26 for the first and hAd5 for the second dose) [44, 45].

All adenovector vaccines express the full-length S protein, but only the Janssen hAd26 vaccine contains stabilizing mutations similar to those engineered into the mRNA vaccines [60]. Interestingly, a recent study of HeLa S3 cells transduced with the ChAdOx1 AstraZeneca vaccine vector showed that the S protein is expressed and transported to the plasma membrane in its native prefusion structure, without any stabilizing mutations [61]. These data suggest that the SARS-CoV-2 S protein may be less labile and prone to switch into its postfusion structure as previously assumed. Indications for relative stability in the absence of mutations were also obtained in recent structural studies of a soluble S protein construct lacking the membrane anchor [62].

## Inactivated whole-virus vaccines

China has spearheaded the development of conventional inactivated whole-virus vaccines, and so far has achieved considerable success with this traditional and long-established technology. Vaccines produced by the companies Sinovac Biotech (Sinovac, Beijing, China) and Sinopharm (Sinopharm, Beijing, China) have been approved for general public use in China, and the Sinopharm vaccine also in Bahrain and the United Arab Emirates, after clinical trials had shown an efficacy of 86% after 2 vaccinations [63]. Mixed reports were obtained for Sinovac’s vaccine termed Coronavac, ranging from 50.4–78% efficacy in a Brazilian trial, 91.25% in Turkey and 65.3% in Indonesia [64]. Since these data are only figures from press releases it is difficult to assess their significance, and differences may be caused by the application of different criteria for calculating rates of protection. For both vaccines, final published results of phase 3 clinical trials are pending.

The current inactivated vaccines from China as well as a vaccine manufactured by Bharat Biotech (Bharat, Hyderabad, Telangana, India) in India and a vaccine in development by the European company Valneva (Valneva, Saint-Herblain, France) [65] are produced by very similar and established technologies. The virus is grown in Vero cells, chemically inactivated, purified more or less extensively and supplemented with adjuvants. In all cases, inactivation is carried out by betapropiolacton (BPL), which has been successfully used for the preparation of other inactivated vaccines, such as rabies vaccines [66].

Some concern was raised through a recent study that analyzed the molecular architecture of BPL-inactivated SARS-CoV‑2, because it revealed that almost all spikes had adopted a postfusion structure [67] (*see* section “The viral spike protein”). In contrast, formalin-inactivated virus preparations display the spike protein primarily in its prefusion conformation [8]. Under certain conditions of preparation, BPL may therefore be a suboptimal choice for inactivation of SARS-CoV‑2, because these vaccines could induce a disproportionately high level of binding but non-neutralizing antibodies at the expense of potently neutralizing antibodies. This unresolved issue may be clarified either by structural analyses of the current inactivated vaccines and/or by determining the ratios of binding and neutralizing antibodies in post-vaccination sera in comparison to other vaccines.

## Subunit vaccines

The COVID-19 candidate vaccine landscape database compiled by the WHO [65] shows that the category of protein subunit vaccines contributes by far the highest number (32%) to the total of 63 candidates currently in clinical development. Only one of those (NVX-CoV2373, developed and manufactured by US company Novavax (Novavax, Gaithersburg, MD, USA) has so far completed phase 3 clinical trials. As recently reported [68], the vaccine was highly effective in preventing disease (89.3%) in a trial conducted in the UK. Substantially lower protection rates were recorded from a trial in South Africa (60.1%), a discrepancy that might be associated with antigenic differences between the predominantly circulating strains B.1.1.7 (N501Y.V1) in the UK and B.1.351 (N501Y.V2) in South Africa. The possible impact of antigenic changes on vaccine efficacy through mutations in the S protein are specifically addressed in the following section.

The antigen used in the Novavax vaccine is a recombinant full-length S protein with stabilizing mutations [69] produced in Sf9 insect cells. Because the protein in its full-length form (Figs. 1, 3 and 4) is not secreted but associated with cellular membranes, it has to be extracted by detergent solubilization and chromatographically purified in an elaborate production process. Nanoparticles of approximately 40 nm size are formed by mixing the purified protein with Novavax’s proprietary Matrix‑M^TM^ adjuvant, composed of saponin from the tree *Quillaja saponaria Molina *as well as cholesterol and phospholipid. Detailed structural analyses indicated that the S protein in the vaccine is stably locked in the preferred prefusion conformation and each nanoparticle is studded with up to 14 spike proteins [70]. Because of its stability, the vaccine can be stored at 4 °C for prolonged periods of time and therefore—like adenovector and inactivated vaccines—offers logistic advantages over the currently authorized mRNA vaccines, which require storage at low-freezing temperatures.

## Impact of new SARS CoV-2 virus variants on vaccines

**Fig. 5:**
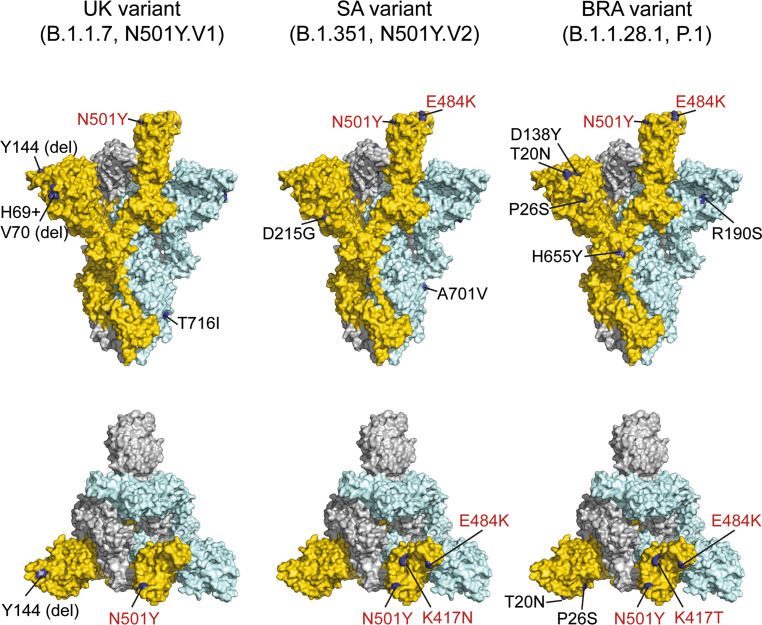
SARS-CoV-2 spike protein with mutations of variants of concern. Side views: upper panels. Top views: lower panels. Surface representation of the spike in its prefusion conformation with one RBD up of the UK, SA, and BRA variants (PDB ID 6ZGG; [11]). Color code of the three S protomers as in Fig. 2. Only surface-exposed mutated amino acids are shown in *dark blue* and labeled (RBD residues in *red*). The mutation P681H in the UK variant is not highlighted, since the loop containing residue 681 is not resolved in the available structures. Mutations in the lower panels are only shown in the S protomer colored in *gold*

Since the appearance and global spread of SARS-CoV‑2, the potential of adaptive mutations was immanent and a concern with respect to vaccine development. Although a plethora of mutations were identified in circulating virus strains over time [71], most of them were unremarkable and the virus appeared to be antigenically stable. Recently, the situation changed through the emergence of virus strains that might be more transmissible but also have mutations at some of the most important antigenic sites of the virus. These variants include the UK strain B.1.1.7 (N501Y.V1), the South African strain B.1.351 (N501Y.V2), and the Brazilian strain B.1.1.28.1 (P.1), which display a varying number of mutations in the S protein (Fig. 5; [71]).

The UK variant has eight mutations in the spike protein, one of them (N501Y) in the immunodominant RBD [1]. At the same exposed site of RBD, the South African variant and the Brazilian variant have two additional mutations (E484K and K417N/K471T, respectively). These two mutations may be especially relevant for antibody recognition, because they cause drastic changes in electrical charges of corresponding epitopes at the protein surface. First data on the comparative neutralization of original and new variants by serum samples obtained after vaccination or infection are consistent with these considerations. The UK mutant was neutralized equally well as the wildtype by postvaccination samples, both after immunization with the Biontech/Pfizer mRNA vaccine [72] and the Moderna mRNA vaccine [73]. In an interim analysis of field efficacy, the Oxford/Astra Zeneca vaccine appeared to be equally protective against B.1.1.7 and canonical lineages, although neutralization titers were ninefold lower [74]. In the Moderna vaccine study [73], substantially reduced neutralization was measured with the South African mutant, although a certain degree of neutralization was still observed with all of the sera from the panel analyzed. Of even more concern are data obtained with the South African mutant and postinfection samples, because not only strong reductions of neutralizing activity but even complete escape from neutralization was observed in several instances with a panel of convalescent plasmas [75]. At the time of this writing, specific data with the Brazilian variant had not yet been published. Based on the nature and location of mutations in S (Fig. 5), however, similar reductions in neutralization can be expected. Recent reports of lower efficacy rates in phase 3 clinical trials of the Novavax subunit vaccine in South Africa than in the UK and the Janssen Adeno26 vaccine in Latin America and South Africa than in the USA (*see* previous section) may reflect the impact of circulating variant strains on vaccine efficacy. Therefore, close monitoring of the emergence and spread of new variants will be essential not only for the aspect of potentially increased transmissibility but also for the risk of escape from vaccine-induced immunity. Disturbingly, the E484K mutation (characteristic of the South African variant) has recently also been detected in B.1.1.7 isolates from the UK, and independent acquisition events have been suggested [76].

## Open questions and future directions

The achievements in developing COVID-19 vaccines are tremendous and unprecedented in the history of medicine. Never have new vaccine technologies been implemented into practical use more rapidly and production capacities for billions of vaccine doses generated so effectively from scratch. A certain degree of excitement if not euphoria is therefore warranted. Nevertheless, not all obstacles may already have been overcome and some questions are pending. One of the most important is of course the issue of antigenic variation, and we are just about to recognize this aspect as a real and not only a theoretical problem. We do not know, however, how prominent it will become and whether a situation of antigenic drift similar to that of influenza virus will develop, requiring continuous update of vaccine compositions. Such adaptations might be most easily achieved with mRNA and vector vaccines. An alternative scenario would be the establishment of a limited number of dominant strains, necessitating the generation of multivalent vaccines. Equally important is the unresolved question of the duration of immunity and whether and at which intervals booster vaccinations will be necessary. Considering the substantially different kinds of vaccines, the question will also arise whether these can be combined in prime-boost schedules and/or exchanged for booster vaccinations. A first clinical study addressing the topic of mixing COVID-19 vaccines has already been launched in the UK [77]. There is an ongoing discussion concerning the question of vaccine-induced herd immunity and whether vaccinated people are still able to transmit the virus on infection, even when they are protected from developing disease. A recently published study on the efficacy of the CHAdOx1 nCoV-19 (AZD1222) vaccine showed that viral burden was strongly reduced after first and second vaccinations, suggesting a potentially strong impact on transmission [46].

After mRNA vaccination, more frequent and more severe side reactions were reported after the second than after the first dose of vaccination [30, 31] and also when given to individuals with a past infection [78], suggesting an effect of specific immunological memory. The mechanisms underlying this phenomenon have not been specifically addressed, but they might be related to the fact that the native S protein as well as processed antigenic peptides in complex with MHCI and/or MHCII molecules are expressed at the surface of mRNA-transduced cells (Fig. 3). Upon booster vaccination, these cells can therefore become a target for cytotoxic immune reactions originating from priming by either vaccination or natural infection [79]. These reactions may involve specific T cells (e.g. tissue-resident memory T cells [80]) and also antibody-mediated mechanisms such as antibody-dependent cellular or complement-mediated cytotoxicity [81]. Why no such increased but rather decreased reactogenicity was observed after the second dose of adenovector vaccination [48, 59], which also results in the expression of viral antigens on target cells (Fig. 4), is currently unknown; however, since mRNA-LNPs are taken up nonspecifically and adenovector particles through specific receptors [43, 82], target cells may differ. This aspect remains to be investigated.

Although an impressive number of COVID-19 vaccines have now been authorized in many countries and used for mass vaccination campaigns, there is still a long list of candidate vaccines in the pipeline. As documented by WHO [65], the number is markedly higher than 200, with 63 in clinical and 174 in preclinical development. Improvements may always be possible, and promising approaches include the development of intranasal vaccines for inducing local immunity [83], self-replicating RNA [33] as well as improved and more thermostable liposome carriers for RNA vaccines [84]. Impressive progress is also made in the area of subunit vaccines that contain only the RBD and use carriers for improving immunogenicity [85–87].

Next generation candidates, however, will face the problem of demonstrating vaccine efficacy in a situation where effective vaccines already exist. Placebo controls (as used in the phase 3 trials of the currently authorized vaccines) will be difficult to perform in such a situation, and the demonstration of noninferiority to existing vaccines will require even more participants and make trials even more expensive. A remedy to such obstacles would be the establishment of a reliable in vitro correlate of protection that is accepted by licensing authorities. For the time being, we can be very satisfied with the current progress and confident that emerging problems will be tackled with similar energy and ingenuity as were required for the breakthroughs already achieved.
